# Structural and magnetic properties of cobalt iron disulfide (Co_x_Fe_1−x_S_2_) nanocrystals

**DOI:** 10.1038/s41598-018-22996-1

**Published:** 2018-03-19

**Authors:** Henrik Gabold, Zhongyue Luan, Neelima Paul, Matthias Opel, Peter Müller-Buschbaum, Matt Law, Amitesh Paul

**Affiliations:** 1 Technische Universität München, Physik-Department, Lehrstuhl für Neutronenstreuung, James-Franck-Strasse 1, D-85748 Garching, Germany; 2 Department of Chemical Engineering & Materials Science, University of California Irvine, Irvine, CA 92697 USA; 3 Heinz Maier-Leibnitz (MLZ), Technische Universität München, D-85748 Garching, Germany; 4 Walther-Meißner-Institut, Bayerische Akademie der Wissenschaften, D-85748 Garching, Germany; 5 Technische Universität München, Physik-Department, Lehrstuhl für Funktionelle Materialien, James-Franck-Str. 1, D-85748 Garching, Germany; 6 Department of Chemistry, University of California Irvine, Irvine, CA 92697 USA

## Abstract

We report on synthesis and investigation of nanocrystalline cobalt-iron-pyrites with an emphasis on nanocrystal structure, morphology and magnetic behavior. The nanocrystals (NCs) were 5–25 nm in diameter as characterized using scanning electron microscopy (SEM) and transmission electron microscopy (TEM). With an increase in Fe fraction, X-ray diffraction and small-angle-X-ray scattering (SAXS) showed a systematic decrease in lattice constant, primary grain/NC size (15 to 7 nm), and nanoparticle (NP) size (70 to 20 nm), respectively. The temperature dependence of the DC magnetization and AC susceptibility versus frequency revealed a number of magnetic phases in Co_*x*_Fe_1−*x*_S_2_. Samples with *x* = 1 and *x* = 0.875–0.625 showed evidence of superspin glass (SSG) behavior with embedded ferromagnetic (FM) clusters of NPs. For *x* = 0.5, samples retained their mixed phases, but showed superparamagnetic (SPM) behavior with antiferromagnetic clusters suppressing magnetic dipolar interactions. Below *x* = 0.5, the pyrites show increasing paramagnetic character. We construct a phase diagram, which can be understood in terms of competition between the various dipolar, exchange, inter- and intracluster interactions. Our results suggest that NC size and shape can be tuned to engineer spin-polarized ferromagnetism of *n*-doped iron pyrite.

## Introduction

3*d* transition metal chalcogenides, in particular metal disulfides such as the pyrites (cubic MS_2_, with M = Mn, Fe, Co, Ni, Cu, Zn, Ru), are of great interest due to their multiple functional electronic and magnetic properties. Iron pyrite (FeS_2_) is a diamagnetic semiconductor (with a band gap of 0.95 eV) of interest for photovoltaics^1^. CoS_2_ is a metallic itinerant ferromagnet with a transition temperature *T*_*C*_ = 120 K^2,3^.

The solid solution Co_*x*_Fe_1−*x*_S_2_ forms a solid solution over the entire compositional range (0 ≤ *x* ≤ 1). In the dilute limit (*x* < 0.01), Co is reported to be a very shallow donor that gives *n*-type FeS _2_ and metallic electrical behavior at low doping levels. This is due to the shift in Fermi level into the conducting band. Co_*x*_Fe_1−*x*_S_2_ is also believed to be paramagnetic at room temperature for all values of *x* > 0^4^. Undoped CoS_2_ and doped alloyed samples for *x* > 0.7 show a ferromagnetic to paramagnetic phase transition at *T*_*C*_ ≈ 120 K^5^. We therefore expected Co_*x*_Fe_1−*x*_ S_2_ nanocrystals (NCs) to exhibit a range of magnetic behavior, from pure diamagnetism (*x* = 0) to fully spin-polarized ferromagnetism (half metallic ferromagnetism) at low temperatures for *x* > 0.5.

The preparation of Co_*x*_Fe_1−*x*_S_2_ NCs offers the possibility of tunable magnetic properties by changing particle size, shape, and surface chemistry via synthesis conditions^6^ or post-synthesis surface treatments^3^. NCs are expected to show strongly enhanced magnetic moments due to their high surface area to volume ratio and the reduced coordination of atoms at the surface. The magnetic properties of Co_*x*_Fe_1−*x*_S_2_ NC powders have not yet been reported.

In order to harmonize the terminologies used in describing different structural entities in the paper we define them at the onset. A crystallite or a grain is defined as a single crystalline domain which we call nanocrystal; a nanoparticle (NP) is defined as an object that may consist of one or more crystallites/grains and lastly, a cluster is a collection of nanoparticles. The polydispersity index of a particle ensemble, which is given by the standard deviation of the particle sizes divided by the mean of their sizes, defines monodispersity. If the index parameter is smaller than 0.25, then the particles are monodispersed^7^.

One may note that different magnetic interactions take place on different length scales, e.g. exchange interaction between NCs and dipole interaction between NPs. In general, nanocrystallites can have random orientation of the anisotropy axes varying locally (random magnetic anisotropy). Due to exchange interactions, however, the particles can be magnetic. Also due to the finite size of the particles one can have stray fields/dipolar fields and, finally, due to the aggregation one can have dipolar interactions between the particles. The transition from ferromagnetism to superparamagnetism (SPM) or superspin glass (SSG) behavior is generally expected for discrete small clusters where the individual magnetic moments within such clusters are thermally unstable. The SSG state is believed to result from the frustration generated by dipole-dipole interactions among superspins (magnetic moments of nanoparticles) and from disorders in the system (e.g., the random distributions of particles, positions, sizes and anisotropy-axis orientations). A further increase of inter-particle interactions may lead to a kind of ferromagnetic domain state or superferromagnetism (SFM)^8^.

Here, we report the synthesis of Co_*x*_Fe_1−*x*_S_2_ NCs and their structure, morphology and magnetic properties. We measured the lattice constant, NC size and NP size as a function of composition *x* using X-ray diffraction (XRD), small angle X-ray scattering (SAXS)^9^ and scanning electron microscope (SEM)/transmission electron microscope (TEM). DC magnetization and AC susceptibility measurements were performed as a function of temperature and frequency. At low temperatures, samples with *x* = 0.625–1.0 appear to consist of ferromagnetic (FM) nanoclusters embedded in a superspin glass matrix. Samples with *x* = 0.5 behave as antiferromagnetic (AF) clusters embedded in a superparamagnetic matrix. Below *x* = 0.5, the samples are predominantly paramagnetic. Thus, the overarching trend in magnetic properties can be seen as a gradual transition between these extremes, going from ferromagnetism in the Co end, via antiferromagnetism to an increasingly paramagnetic behavior for high Fe content. All these have lead to a fairly complex magnetic phase diagram which is explained in terms of competition between the various dipolar, exchange, inter- and intracluster interactions.

## Results and Discussions

### SEM and TEM

**Figure 1:**
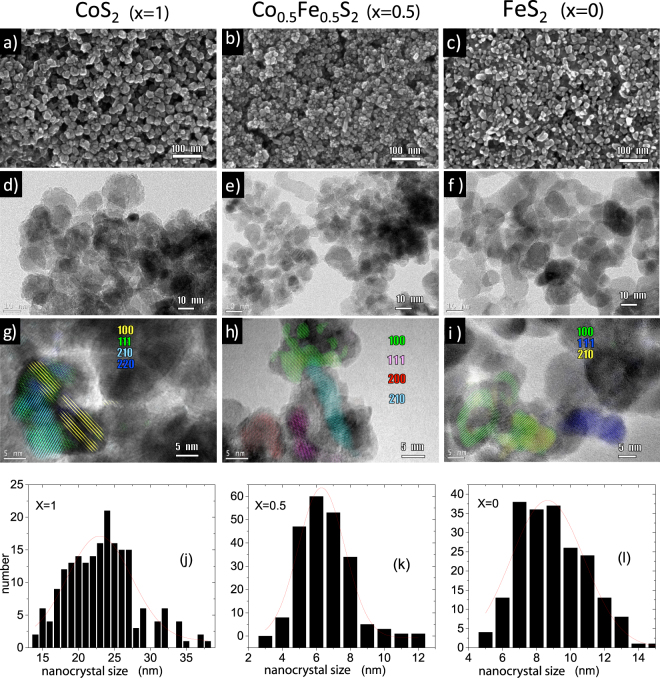
SEM and TEM imaging. (**a–c**) SEM and **(d–i)** TEM images of representative Co_*x*_Fe_1−*x*_S_2_ NC samples with *x* = 1, 0.5 and 0. The lattice-resolved TEM images **(g–i)** are color coded according to lattice plane. **(j–l)** Size histograms from analysis of the SEM images. According to the imaging statistics, the mean diameters of 24 ± 5 nm (*x* = 1), 6.0 ± 1.5 nm (*x* = 0.5) and 9.0 ± 2.5 nm (*x* = 0) are in good agreement with the values estimated from the Gaussian fittings of the size distribution diagrams 23.0 ± 5.3 nm (*x* = 1), 6.3 ± 1.6 nm (*x* = 0.5) and 8.6 ± 2.5 nm (*x* = 0).

SEM and TEM provide real space images of the NCs. Figure 1(a–c) shows the SEM images of typical samples with nominal composition *x* = 1.0, 0.5 and 0.0. The NCs are irregular in shape, fairly polydisperse, and somewhat aggregated. From the TEM images in Fig. 1(d–f), we estimate that the NCs have an average diameter of 24 ± 5 nm (*x* = 1), 6.0 ± 1.5 nm (*x* = 0.5) and 9.0 ± 2.5 nm (*x* = 0). High-resolution TEM (HRTEM) images in Fig. 1(g–i) show the lattice planes of the samples. The observed lattice-fringes correspond to the {100}, {111}, {200}, {210} and {220} planes of pyrite Co_*x*_Fe_1−*x*_S_2_. Size histograms compiled from the SEM data are shown in Fig. 1(j–l). Analysis of the Gaussian fits gave an estimate of the mean NC size. The average polydispersity index in our samples is around 0.35. Statistical processing of the raw TEM data were exported and plotted using Origin 9.0 software.

### XRD

**Figure 2:**
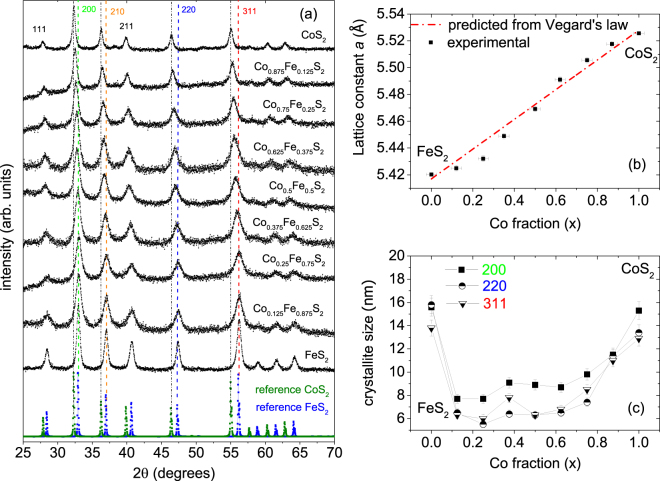
Powder XRD measurements. (**a**) pXRD patterns for Co_*x*_Fe_1−*x*_S_2_ NCs, with *x* = 0.0, 0.125, 0.25, 0.375, 0.5, 0.625, 0.75, 0.875, and 1.0. The plots for different composition (*x*) are y-offset for clarity. The reference pXRD patterns of cattierite (CoS_2_: PDF 00-041-1471) and iron pyrite (FeS_2_: PDF 00-042-1340) are shown below without an offset. The dashed vertical lines indicate the positions of the FeS_2_ 200, 210, 220, and 311 reflections. (**b**) Extracted cubic lattice constants for the different samples. Vertical error bars denote the uncertainty in the Rietveld fits to a cubic pyrite structure. The horizontal error bars indicate the compositional deviations following the Vegard’s law from the nominal ones. (**c**) Plots of crystallite size versus *x* as estimated from the Rietveld refinements of the XRD patterns corresponding to the 200, 220, and 311 reflections.

XRD and SAXS are indirect methods, but they provide statistical information as opposed to local information from SEM or TEM. Figure 2(a) shows the typical XRD data for the Co_*x*_Fe_1−*x*_ S_2_ NC powders. The samples are apparently phase pure within the limit of detection of this technique. A gradual shift in the peak positions towards lower 2*θ* values with increasing *x*, indicates a systematic increase in the lattice constant, as expected for Co_*x*_Fe_1−*x*_S_2_ solid solutions. The dotted vertical lines show the reference peak positions for pure FeS _2_ (*x* = 0) and pure CoS _2_ (*x* = 1). The diffraction peaks at the two ends are consistent with the values given in the standard PDF cards for FeS_2_ (PDF 00-042-1340), CoS_2_ (PDF 00-041-1471).

The patterns were analyzed by Rietveld refinement^10^ using the Highscore Plus software (v4.1, PANalytical B.V., Almelo, Netherlands)^11^. The extracted lattice constants have been plotted in Fig. 2(b) along with the predicted lattice constant (*a*), following Vegard’s law^6^. Note that we have included the binary end members 5.417 Å for FeS _2_ and 5.528 Å for CoS_2_. The estimated accuracy in composition is ±6%. We have used the lattice constants to estimate the NC composition, which is in good agreement with the nominal one.

We also directly measured the Co:Fe ratio of each sample using energy dispersive spectroscopy (EDS) mapping in a TEM. The measured (expected) values of *x* averaged over thousands of NCs across several areas of each sample were 0.0 (0.0), 0.140 ± 0.014 (0.125), 0.270 ± 0.027 (0.250), 0.340 ± 0.013 (0.375), 0.380 ± 0.015 (0.500), 0.600 ± 0.087 (0.625), 0.740 ± 0.032 (0.750), 0.880 ± 0.017 (0.875), and 1.0 (1.0). These values agree quite well with the XRD results and demonstrate that the average composition of the Co_*x*_Fe_1−*x*_S_2_ NCs is close to the intended composition. High-resolution EDS mapping revealed significant compositional variation from NC to NC in some of the samples, but poor sample stability under the focused electron beam made accurate quantification of the degree of particle-to-particle inhomogeneity very difficult, so it was not pursued further.

Figure 2(c) shows the crystallite size as obtained from the Rietveld refinements corresponding to the 200, 220 and 311 reflections. The crystallite size decreases from 14 ± 2 nm at *x* = 1.0 to 7 ± 1 nm for *x* = 0.125 to 0.625, then increases gradually to 15 ± 1 nm for *x* = 0.0. A similar trend was observed following the evolution of all main peaks. Alternatively, strain-free crystallite size was determined from individual peak widths using the Scherrer formula which give similar values. The size of the crystallites is fairly similar to that observed by TEM. However, their size is smaller than the size of the NPs observed by SAXS, showing that some of the NPs are composed of a few NCs.

### SAXS

The basic theory of SAXS applied in this work involves the determination of a set of parameters related to several average values of the radii of the NPs. For polydisperse systems the local monodisperse approximation (LMA) is commonly used^12,13^. In the present work, we have considered scattering from spheres of two different sizes of radii R and R′, with ΔR and ΔR′ representing the mean of the particle radii and their respective distributions, as this provides the most accurate description of the form factor for the present data. The corresponding inter-particle distances (*ξ* and *ξ*^′^ and their respective standard deviations *σ* and *σ*′) are related to the pair correlation function embedded within the scattering cross-section^14,15^.

**Figure 3:**
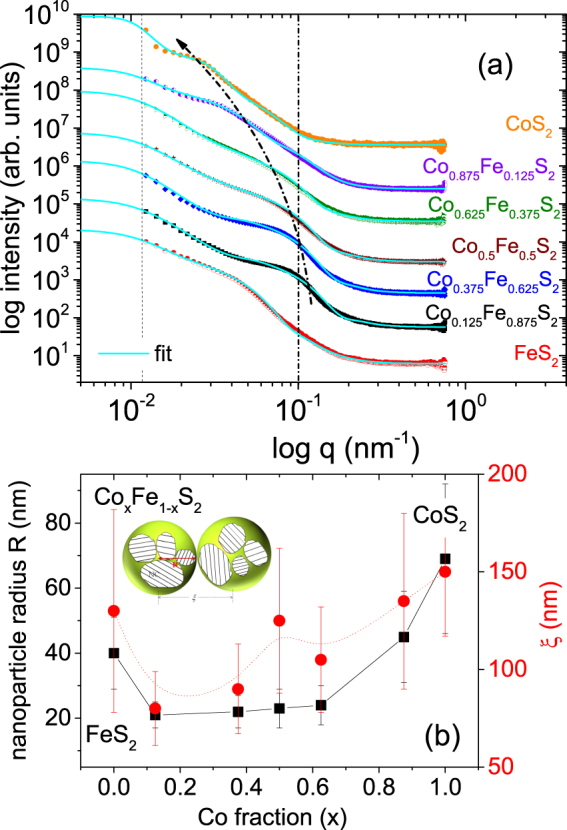
SAXS measurements of NC powders. (**a**) 1-D SAXS profiles of some Co_*x*_Fe_1−*x*_S_2_ NC powders plotted as intensity versus scattering vector *q* where *x* = 0.0, 0.125, 0.375, 0.5, 0.625, 0.875 and 1.0 along with their fits within the LMA. The curved arrow, corresponding to R, acts as a guide to the eye for the gradual shift of the intensity maxima from *q* = 0.12 nm^−1^ to *q* = 0.02 nm^−1^ with *x*. The dotted straight line at *q* = 0.1 nm^−1^, represents the characteristic *q* position of the lower intensity maxima corresponding to R′. The instrument resolution limit indicated by a second dotted line is reached below *q* = 0.01 nm^−1^. (**b**) Nanoparticle radius R and inter-particle distance *ξ* as a function of NC composition. Inset shows an illustration of the NCs and NPs.

In Fig. 3(a), the scattered intensity is plotted as a function of the total scattering vector *q*. From the 1-D data, one can see that all samples show a prominent scattering feature in the form of a hump that shifts from 0.12 nm^−1^ to 0.02 nm^−1^ as composition changes from *x* = 0.125 to *x* = 1. This feature appears at 0.05 nm^−1^ for FeS_2_. The shift of the hump with *x* can also be interpreted as a change in NP assemblies with different inter-particle correlations. An additional smaller hump-like feature is seen at 0.1 nm^−1^ for all compositions. Below *q* = 0.01 nm^−1^, the instrument resolution limits us to make a proper estimate of NP size.

Figure 3(b) shows the two parameters (NP radius R and inter-particle distance *ξ*) as a function of *x*. R is 40 nm for *x* = 0. R decreases to 20 nm and then increases to 70 nm with increasing *x*, while R′ always remains around 30 nm. It may be noted that the trend in NP size (particle diameter 2 R) is similar to that of the NC size extracted from the XRD/TEM data. This signifies that the ratio of the NC size and NP size is constant at around six to eight. This indicates that each NP contains a similar number of NCs, regardless of its composition. The inter-particle distances *ξ* maintain a small variation from 80 ± 20 nm to 150 ± 33 nm within the LMA. One may note that the SEM/TEM data cannot be directly correlated to the SAXS data where the accessible length scale depends upon the scattering geometry. The former is a local probe whereas the latter has more sampling statistics, giving information on the statistically averaged value of sample morphology (object geometry, size, size distribution and spatial correlations) over a much larger sample volume. Notably, from TEM one gets a number-weighted mean of the NC/NP size but with XRD it is volume-weighted and with SAXS it is intensity-weighted.

### Magnetization

#### FC and ZFC

**Figure 4:**
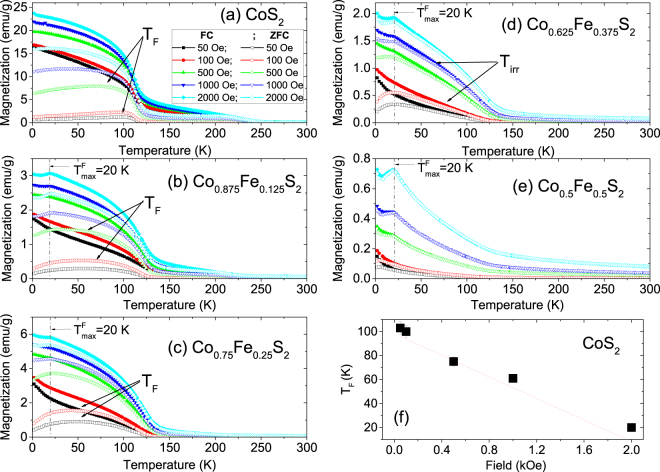
FC and ZFC measurements. (**a–e**) The temperature dependence of the DC magnetization as measured for Co_*x*_Fe_1−*x*_S_2_, where *x* = 1, 0.875, 0.75, 0.625 and 0.5. The measurements were done on heating at various fields (starting from 50 Oe to 2000 Oe) after zero field cooled (ZFC) and field cooled (FC) in 70 kOe. A well defined broad maximum can be observed for the ZFC curves (*T*_F_) followed by a furcation point (*T*_irr_) with the FC curves for some compositions above *x* = 0.5. (**f**) Plot of *T*_F_ versus field for *x* = 1.

To characterize the magnetic properties of the NCs, the magnetization (*M*) was measured as a function of temperature (T). We used five different applied fields *H* of 50, 100, 500, 1000 and 2000 Oe during measurements with increasing temperature. The samples were initially cooled down to 5 K either in the presence of *H* = 70 kOe (FC) or with no magnetic field (ZFC). The *M*(T) curves are shown in Fig. 4(a–e).

For *x* = 1, 0.875, and 0.75, the ZFC curves show a broad maximum (particularly at lower fields) which can be referred to as blocking/freezing temperature (*T*_F_) of the polydisperse NCs, while the FC curves do not show a temperature independent platform-like behavior at low temperatures. The appearance of the maximum in the ZFC curve suggests that the assembly of magnetic nanoparticles passes from the blocked/frozen state to the superparamagnetic regime as the temperature rises. The broadness of the peak is due to the distribution of blocking temperatures, which is expected from a system with particle size distribution. The FC curves continue to increase gently with decreasing temperature for *x* ≥ 0.75, and steeply for *x* ≤ 0.625. This strong history dependence is a generic feature of several commonly known magnetic systems like superspin glasses and superparamagnets^16^. In Fig. 4(f), we show a gradual decrease of *T*_F_ with field for *x* = 1, which suggests that the frozen state is suppressed by the field.

We find a eight-fold decrease in magnetization from *x* = 1 to *x* = 0.875 which can be plausibly due to an initial disorder in the system^17^. This is followed by an increase in magnetization for *x* = 0.75 which is not understood at present. On further increase in Fe content, a monotonous decrease in magnetization can be seen at least up to *x* = 0.5. With increase in Fe below *x* = 0.5, the system becomes increasingly paramagnetic as the *M*(*H*) hysteresis curve at *x* = 0.5 still displays a positive slope.

We also observe a maximum in *M*(T) at around ${T}_{{\rm{\max }}}^{F}$ = 20 K for *x* = 0.875 to 0.5, also known as spin freezing temperature, which is a typical feature of systems with antiferromagnetic or ferrimagnetic ordering and thus indicates that a small proportion of Co oxide is present in every sample. These ordering temperatures have often been seen to decrease with nanocluster size^18^. Since the magnetization of CoS _2_ is higher than other compositions, the ${T}_{{\rm{\max }}}^{F}$ peak remains in the background. The origin of ${T}_{{\rm{\max }}}^{F}$ was further verified by fully oxidizing the CoS_2_ sample in air, after which a single sharp peak was identified at 20 K in the susceptibility plot.

**Figure 5:**
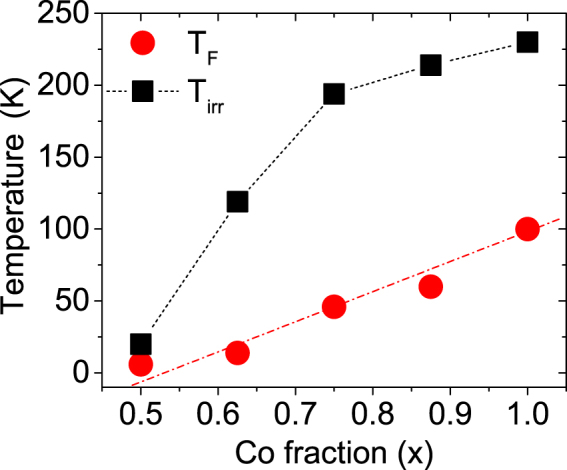
Plot of *T*_F_ and *T*_irr_ with *x*. Plot of furcation temperature in FC-ZFC curves *T*_irr_, broad maximum in ZFC curve *T*_F_ for Co_*x*_Fe_1−*x*_S_2_ as a function of *x*, where *x* = 1, 0.875, 0.75, 0.625 and 0.5. The increase in *T*_F_ and *T*_irr_ can be correlated to the increase in dipolar interaction.

A well-defined irreversibility temperature (*T*_irr_), i.e. the temperature where the FC and ZFC curves diverge, could be identified. The irreversibility temperature corresponds to the supermagnetic transition of the biggest nanoparticles in the assembly. We plot *T*_F_ and *T*_irr_ versus *x* in Fig. 5. Both, *T*_F_, *T*_irr_, are found to increase with *x* or in other words they scale with particle radius and inter-particle distance. Linear scaling of *T*_F_ signifies an increase in the anisotropy energy barrier^19^. *T*_irr_ shows a gradual saturation behavior with *x*, which signifies a progressive blocking/freezing of bigger nanoparticles^20^.

**Figure 6:**
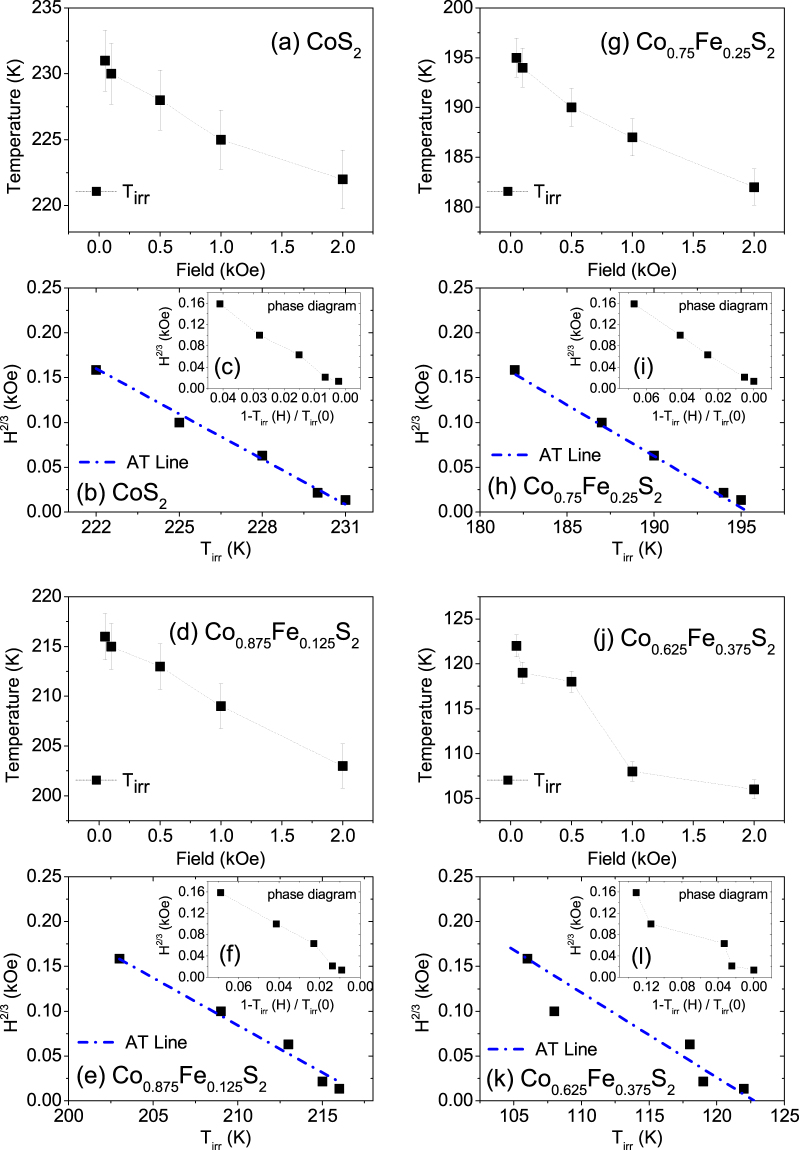
Plot of *T*_irr_ with field. (**a**,**d**,**g**,**j**) Plot of furcation temperature in FC-ZFC curves *T*_irr_ as a function of field for Co_*x*_Fe_1−*x*_S_2_, where *x* = 1, 0.875, 0.75 and 0.625. (**b,h,e,k**) Plot of *H*^(2)/(3)^ vs *T*_irr_ and its fit following the AT-line. (**c,i,f,l**) Inset shows the phase diagram of the same in a *H*-T plot depicting the boundary line between SSG (below) and SPM (above) phases.

We plot *T*_irr_ versus field only for samples with *x* = 1, 0.875, 0.75 and 0.625 as shown in Fig. 6(a,d,g,j). For sample with *x* = 0.5, a field dependence could not be ascertained. The observed shift of *T*_irr_ to lower temperatures with increasing field can follow the Almeida-Thouless (AT) line that indicates super-spin-glass (SSG) behavior, as shown in Fig. 6(b,e,h,k). The AT line expression is

1 $$H/{\rm{\Delta }}J\propto {(1-\frac{{T}_{irr({\bf{H}})}}{{T}_{irr(0)}})}^{\frac{3}{2}}$$

where *T*_irr(0)_ is the zero field freezing temperature and Δ*J* is the width of the distribution of exchange interactions. Figure 6(c,f,i,l) shows the evolution of *T*_irr_ which can be mapped on the *H*−T plane in order to distinguish the SSG phase from the SPM phase.

#### Hysteresis loop

**Figure 7:**
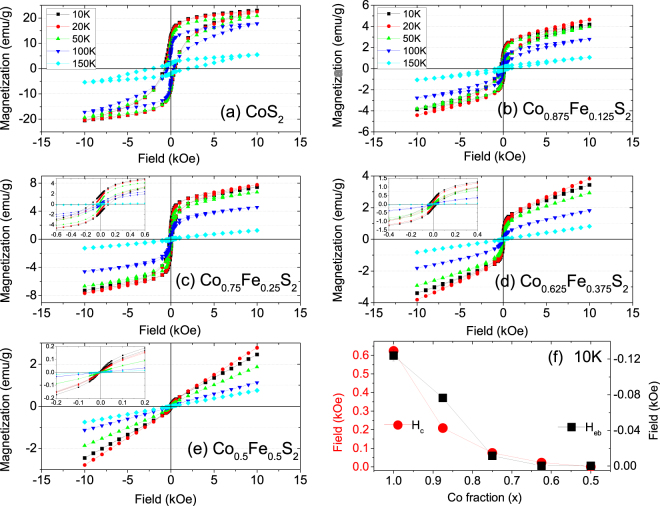
*M*-*H* loop measurements. (**a–e**) The temperature dependence of the hysteresis loops for Co_*x*_Fe_1−*x*_S_2_, where *x* = 1, 0.875, 0.75, 0.625 and 0,5. The insets of (**c**–**e**) show the same within reduced field ranges for clarity. (**f**) Plot of coercive field *H*_c_ and exchange bias field *H*_eb_ at 10 K as a function of *x*.

In-plane *M*-*H* curves of samples with *x* = 1.0, 0.875, 0.75, 0.625 and 0.5 measured at 10–150 K are shown in Fig. 7(a–e). For all temperatures, we find that the magnetization increases with increasing *x*, except for *x* = 0.875, where we find a decrease. This decrease is consistent with the decrease in the FC-ZFC curves discussed in the previous section. The magnetization decreases from *x* = 0.75 onwards and finally disappears below *x* = 0.5. Similar behavior was reported for Co_*x*_Fe_1−*x*_S_2_ single crystals by Leighton *et al*.^5^. Samples with *x* < 0.5 show increasing paramagnetic response. The magnetization of all samples is unsaturated at even the highest fields explored here (10 kOe). We observe ferromagnetic hysteresis loops with coercive fields as large as 0.6 kOe (*x* = 1, T = 10 K). The hysteresis diminishes with increasing temperature and decreasing *x*; for *x* = 0.5, the magnetization exhibits barely any hysteresis even at 10 K. The absence of saturation combined with pronounced hysteresis loops for *x* > 0.5 is characteristic of ferromagnetic grains embedded in SSG-like matrices^8,21^.

**Figure 8:**
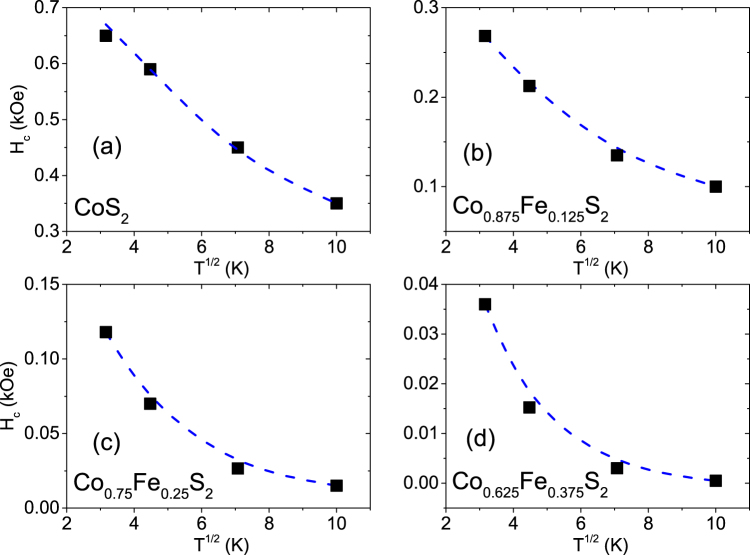
*H*_*c*_versus *T*^1/2^. (**a–d**) The plot of *H*_*c*_ versus *T*^1/2^ for Co_*x*_Fe_1−*x*_S_2_, where *x* = 1, 0.875, 0.75 and 0.625. The dotted curves are a guide to the eye.

We define *H*_*c*_ = (${H}_{c}^{+\alpha }$ − ${H}_{c}^{-\alpha }$)/2 and *H*_*eb*_ = (${H}_{c}^{+\alpha }$ + ${H}_{c}^{-\alpha }$)/2, where ${H}_{c}^{+\alpha /-\alpha }$ are the coercive fields for the positive and negative field axes. We plot the coercivity (*H*_c_) and exchange bias field (*H*_eb_) at 10 K as a function of *x* in Fig. 7(f)). The existence of *H*_eb_ (=−0.12 kOe for *x* = 1) confirms the presence of a small proportion of antiferromagnetic Co oxide. The magnitude of both parameters gradually increases with increasing *x* because the spin-spin coupling is proportional to the concentration of cobalt centers in the lattice which affects the exchange coupling of Co with cobalt oxide. Plots of *H*_*c*_ versus *T*^1/2^ in Fig. 8(a–d)) for *x* = 1, 0.875, 0.75 and 0.625 do not intercept the T axis as expected for the SPM type of non-interacting nanoparticle ensemble, thus indicating a SSG type of behavior in these samples. A linear slope would have indicated a SPM type of behavior.

#### AC susceptibilities

AC magnetic susceptibility (*χ*′(T) = d*M*/d*H*) measurements are utilized in supermagnetic systems due to their ability to probe different values of the relaxation time that categorize paramagnetic or glassy behaviors. To distinguish between SPM and SSG behavior, we measured the temperature dependence of the AC magnetic susceptibility since the frequency response of the peak in the *χ*′(T) curve is different for the SPM phase from the SSG phase. Data were taken over a frequency range of 10 to 10,000 Hz in the presence of a small AC field of 5 Oe and without any DC field. We categorize the data analysis section into three separate ones viz. (i) with *x* = 1.0, (ii) *x* = 0.875, 0.75, 0.625 and (iii) *x* = 0.5.

**Figure 9:**
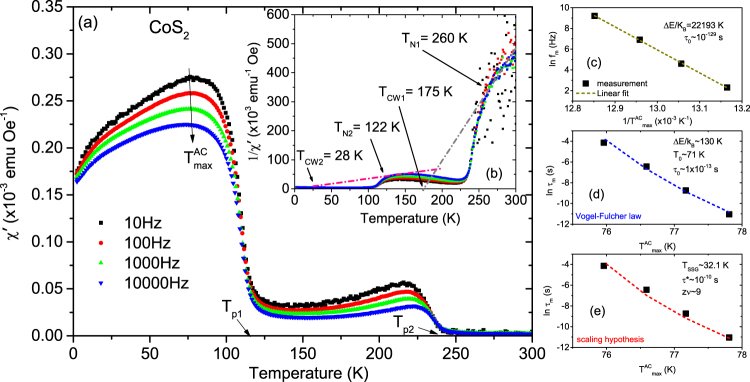
AC susceptibility measurements with *x* = 1. (**a**) The temperature dependence of the real part of the AC susceptibility (*χ*′(T)) for frequencies ranging from 10 to 10,000 Hz for Co_*x*_Fe_1−*x*_S_2_, where *x* = 1. (**b**) Inverse susceptibility versus temperature and its linear fit (gray and pink lines). Frequency dependence of ${T}_{{\rm{\max }}}^{AC}$ and its fit using the (**c**) Néel-Arrhenius model, (**d**) Vogel-Fulcher model and (**e**) the critical slowing down model.

(i) Co_*x*_Fe_1−*x*_S_2_ with *x* = 1.0:

The frequency dependence of the real part of the susceptibility (*χ*′(T)) of a sample with *x* = 1.0 shows two different temperature regimes demarcated by the two paramagnetic Curie temperatures *T*_p1_ = 233 K and *T*_p2_ = 110 K in Fig. 9(a). In each regime, two broad peaks centered at $${T}_{{\rm{\max }}}^{AC}$$$$\simeq $$ 75 K and 225 K are visible. Multiple peaks in *χ*′(T) often indicate the presence of several magnetic phases in a sample. We believe that the two phases, primary and secondary, correspond to the two different magnetic nanoparticle correlations observed in the SAXS measurements described above. One may recall the two humps in the SAXS spectra (Fig. 3(a)), one that shifts with *x* (particularly for *x* > 0.625) and the other one that remains unchanged. The difference in the *χ*′(T) signals, corresponding to *T*_p1_ and *T*_p2_, defines their respective proportionality within the system for a particular composition.

Inverse *χ*′(T) plot is a good marker for the critical temperature to show the differences between, e.g., ferromagnetism and antiferromagnetism below the respective ordering temperature. Above the ordering temperature, its linearity represents a typical Curie behavior. The first part of the magnetic phases in Fig. 9(b) follow a linear behavior according to the Curie-Weiss law. Accordingly, we find two Curie-Weiss temperatures (*T*_CW1_ = 175 K and *T*_CW2_ = 28 K) from linear fits to the inverse *χ*′(T) plot. We have used the increasing portion of 1/*χ*′(T) following *T*_N2_ in determining the average *T*_CW2_ value before it flattens out. For *T*_CW1_, a linear fit to the increasing proportion of the same following *T*_N1_ is more obvious. The positive *T*_CW_ values indicate ferromagnetic interactions between embedded magnetic nanoparticles in a SPM or SSG matrix. In the second part of the magnetic phases, the data depart progressively away from linearity. The points where the hyperbola intersects the temperature axis are the estimated values of *T*_p1_ and *T*_p2_. The deviations from linearity give the corresponding Néel temperatures *T*_N1_ = 260 K and *T*_N2_ = 122 K. Above these temperatures, the magnetic nanoparticles behave according to the Curie-Weiss law, while below where the experimental data deviate from the fit, SPM/SSG-like interactions may determine the response of the system. We have used *T*_N_ to mark the ferromagnetic Curie temperature following its familiarity with the localized-moment picture proposed by Néel in his theory of antiferromagnetism.

Our focus will mainly be on the ${T}_{{\rm{\max }}}^{AC}$ at lower temperature because the higher temperature maxima can be neglected. Note the difference in the ${T}_{{\rm{\max }}}^{AC}$ signals at the two temperatures. This difference signifies a lower proportionality of the secondary phase as compared to the primary phase. The ${T}_{{\rm{\max }}}^{AC}$ peaks decrease in magnitude and shift to higher temperature with higher driving frequency. The shift gives the activation energy and is characteristic of SPM-type or SSG-type behavior. Since a shift in *χ*′(T) peak with frequency is expected for both superparamagnets and superspin glasses, further analysis is need to identify. One may note that non-interacting SPM clusters should show larger frequency dependence than SSG clusters since the distribution of relaxation times is characteristic for the spin-glass phase. We performed several cross checks to qualitatively analyze the dynamical behavior. Three common models of superparamagnetic dynamics are embodied in the (a) Néel-Arrhenius law, (b) Vogel-Fulcher law and (c) power law in addition to the (d) empirical equation, which are all employed in discerning the two magnetic phases^22–26^.

**Figure 10:**
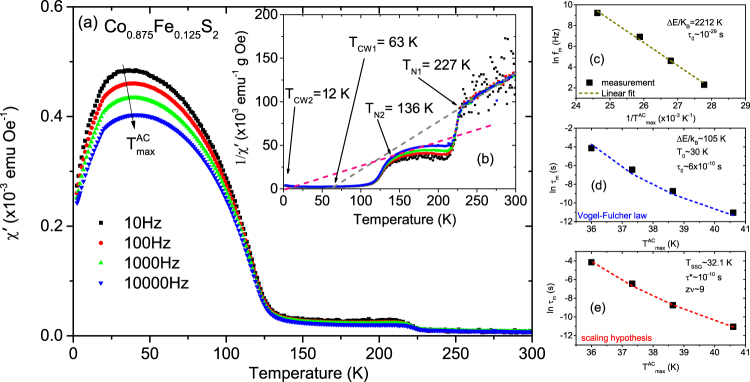
AC susceptibility measurements with *x* = 0.875. (**a**) The temperature dependence of the real part of the AC susceptibility (*χ*′(T)) for frequencies ranging from 10 to 10,000 Hz for Co_*x*_Fe_1−*x*_S_2_, where *x* = 0.875. (**b**) Inverse susceptibility versus temperature and its linear fit (gray and pink lines). Frequency dependence of ${T}_{{\rm{\max }}}^{AC}$ and its fit using the (**c**) Néel-Arrhenius model, (**d**) Vogel-Fulcher model and (**e**) the critical slowing down model.

**Figure 11:**
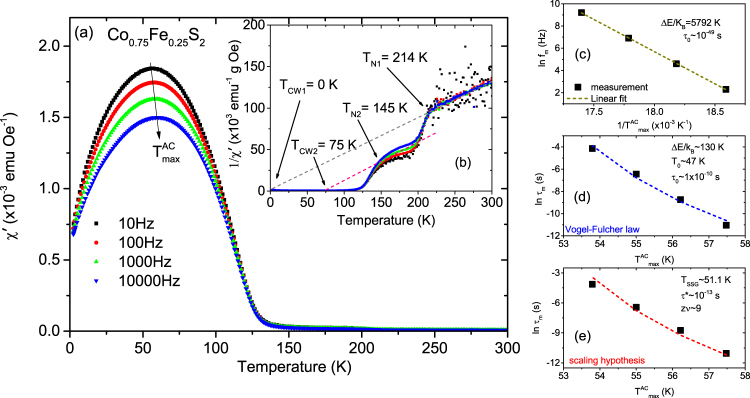
AC susceptibility measurements with *x* = 0.75. (**a**) The temperature dependence of the real part of the AC susceptibility (*χ*′(T)) for frequencies ranging from 10 to 10,000 Hz for Co_*x*_Fe_1−*x*_S_2_, where *x* = 0.75. (**b**) Inverse susceptibility versus temperature and its linear fit (gray and pink lines). Frequency dependence of ${T}_{{\rm{\max }}}^{AC}$ and its fit using the (**c**) Néel-Arrhenius model, (**d**) Vogel-Fulcher model and (**e**) the critical slowing down model.

**Figure 12:**
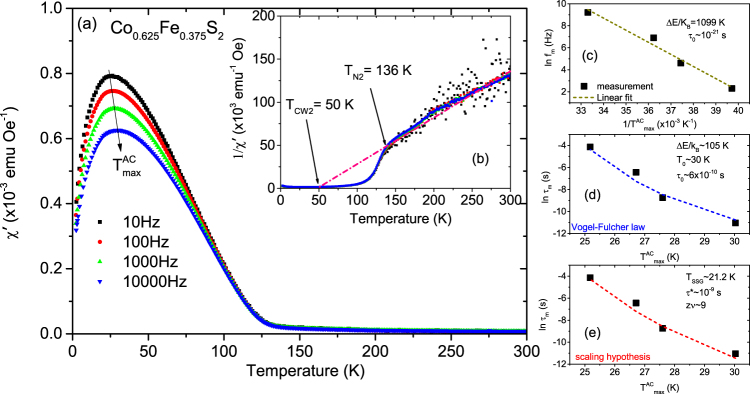
AC susceptibility measurements with *x* = 0.625. (**a**) The temperature dependence of the real part of the AC susceptibility (*χ*'(T)) for frequencies ranging from 10 to 10,000 Hz for Co_*x*_Fe_1−*x*_S_2_, where *x* = 0.625. (**b**) Inverse susceptibility versus temperature and its linear fit (pink line). Frequency dependence of ${T}_{{\rm{\max }}}^{AC}$ and its fit using the (**c**) Néel-Arrhenius model, (**d**) Vogel-Fulcher model and (**e**) the critical slowing down model.
(iii) Co_*x*_Fe_1−*x*_S_2_ with *x* = 0.5:

- Noninteracting SPM clusters generally follow the Néel-Arrhenius law.
  2 $${\tau }_{m}={\tau }_{0}\exp \frac{{\rm{\Delta }}E}{{k}_{B}T}$$
  where *τ*_0_ = (2*π*f_0_)^−1^ is the relaxation time or the inverse of the attempt frequency (*f*_0_) and *τ*_m_ = (2*π*f_m_)^−1^ is the measuring time (~10^2^s for the DC magnetization measurement) or the inverse of the measuring frequency (*f*_m_). ΔE( = K_A_V) is the anisotropy energy or activation energy for cluster formation. Here K _A_ is the anisotropy constant, *k*_B_ ( = 1.38 × 10^−16^ erg/K) is the Boltzmann constant and *V* the average cluster volume critical for SPM or SSG state at *T*_F_. *τ*_0_ has a typical value of 10^−9^–10^−13^ s for SPM behavior.
  We plot the dependence of (${T}_{{\rm{\max }}}^{AC}$)^−1^ on the natural logarithm of the measurement frequency in Fig. 9(c). A linear fit yields a slope of ΔE/*k*_B_ = 22,193 K and this value derives an unreasonably small value of *τ*_0_~10^−129^ s. Based on the unphysical value of *τ*_0_, we conclude that the Néel-Arrhenius expression fails to describe the system, suggesting that magnetic dipole-dipole interactions between the magnetic nanoparticles are important and cannot be ignored.
- The Vogel-Fulcher law phenomenologically describes the frequency response of the relaxation time by taking into account the weak interactions among spin clusters and is expressed by
  3 $${\tau }_{{\rm{m}}}={\tau }_{{\rm{0}}}\exp [\frac{{\rm{\Delta }}E}{{k}_{{\rm{B}}}({T}_{{\rm{\max }}}^{AC}-{T}_{{\rm{0}}})}]$$
  where *T*_0_ is the characteristic temperature that accounts for the static interaction field of the surrounding clusters. We show a plot of ln(*τ*_*m*_) versus ${T}_{{\rm{\max }}}^{AC}$ in Fig. 9(d). The values obtained from a fit to the equation 3 are Δ*E*/*k*_*B*_ ~130 K, *T*_0_ ~71 K and *τ*_0_ ~3 × 10^−13^ s. These values are physically reasonable and comply with the SSG behavior for which the typical value of *τ*_0_ ~10^−10^–10^−13^ s^23^. Thus we believe that the maxima observed in *χ*′(T) correspond to the freezing of weakly interacting NC moments.
- The power law or the scaling hypothesis supposes the existence of an equilibrium phase transition. It relies on the relation of critical slowing down of the relaxation time near the transition temperature. The relaxation behavior is expressed by
  4 $${\tau }_{{\rm{m}}}={\tau }_{\ast }{[\frac{{T}_{{\rm{\max }}}^{AC}-{T}_{SSG}}{{T}_{SSG}}]}^{-z\nu }$$
  Here, τ_*_ is a relaxation time for each nanocluster, *zν* is the dynamical scaling–critical exponent constant^25^ related to the correlation length *ξ*, which is a measure of the size of the lateral coarsening (*ν* describes the divergence while *z* is involved in the dynamical scaling hypothesis *τ*_*m*_ ~ *ξ*^*z*^). In Fig. 9(e) we plot the variation of *τ*_*m*_ (in log scale) versus ${T}_{{\rm{\max }}}^{AC}$. The fit to the equation 4 yields a value of *zν* ~ 9 and τ_*_ ~ 10^−10^ s, which are comparable with the typical values reported for SSG systems^26^.
- Beyond the three models described above, another simple, useful and sensitive criterion to distinguish between the freezing and the blocking processes is to determine the relative shift of the *χ*′(T) peak with frequency using the empirical equation

5 $$p=\frac{{\rm{\Delta }}{T}_{{\rm{\max }}}^{AC}}{\overline{{T}_{{\rm{\max }}}^{AC}}{{\rm{\Delta }}\mathrm{log}}_{10}({f}_{m})}$$

where $\overline{{T}_{{\rm{\max }}}^{AC}}$ is the mean value of the frequency dependent maximum in *χ*′(T), while ${\rm{\Delta }}{T}_{{\rm{\max }}}^{AC}$ is the difference in ${T}_{{\rm{\max }}}^{AC}$ over the frequency interval Δlog_10_(*f*_*m*_^)23,26^. Typically, the parameter p assumes values of 0.0045–0.06 (for SSG phases) and 0.10–0.13 (for SPM phases). In our case, p = 0.014, which again indicates an interacting SSG-type of behavior^22^.

(ii) Co_*x*_Fe_1−*x*_S_2_ with *x* = 0.875, 0.75 and 0.625:
  Figures 10(a), 11(a) and 12(a) plot *χ*′(T) at different frequencies for samples with *x* = 0.875, 0.75 and 0.625. All samples show a broad peak ${T}_{{\rm{\max }}}^{AC}$ in *χ*′(T). Two Weiss temperatures *T*_CW1_ and *T*_CW2_ can be extracted from the linear fits of each inverse *χ*′(T) plot and two deviations from linearity for the inverse *χ*′(T) with two corresponding ordering temperatures *T*_N1_ and *T*_N2_ for *x* = 0.875 and 0.75 as shown in Figs 10(b), 11(b), respectively. For *x* = 0.625, in Fig. 12(b), we find only one *T*_CW2_ and the corresponding *T*_N2_. One may note that the net magnetization in our system largely shows a decreasing trend with decreasing *x*. For *x* = 1, for example, the magnetization is highest and we can see the two peaks in the AC spectra. The secondary peak being much weaker as compared to the primary one shows a systematic decrease with decreasing *x*. Beyond *x* = 0.625, it simply goes into the background. All Curie-Weiss temperatures are positive. Their frequency dependence is typical of SSG behavior as demonstrated using the three models described above and shown in Figs 10(c–e), 11(c–e) and 12(c–e). These are typical signatures of a mixed-phase situation with embedded FM clusters in SSG matrices^27^.

**Figure 13:**
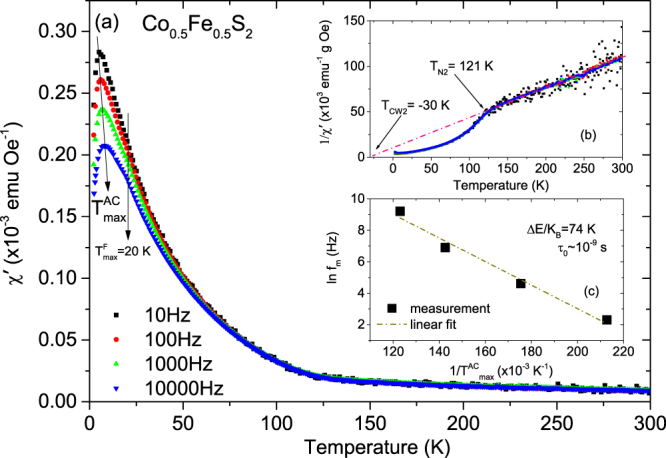
AC susceptibility measurements with *x* = 0.5. (**a**) The temperature dependence of the real part of the AC susceptibility (*χ*'(T)) for frequencies ranging from 10 to 10,000 Hz for Co_*x*_Fe_1−*x*_S_2_, where *x* = 0.5. (**b**) Inverse susceptibility versus temperature and its linear fit (pink line). Frequency dependence of ${T}_{{\rm{\max }}}^{AC}$ and its fit using the (**c**) Néel-Arrhenius model.

The frequency dependence of *χ*′(T) for samples with *x* = 0.5 is shown in Fig. 13(a). A peak (${T}_{{\rm{\max }}}^{AC}$) is observed at ~5 K for all frequencies. The peak shifts to higher temperature and lower height with driving frequency. Plots of inverse *χ*′(T) versus temperature yield a negative Curie-Weiss temperature *T*_CW2_ = −30 K, indicating predominant AF ordering and a deviation from linearity gives *T*_N2_ = 121 K as shown in Fig. 13(b). We performed a similar qualitative analysis for the dynamical behavior. We plot the natural logarithm of the measurement frequency versus (${T}_{{\rm{\max }}}^{AC}$)^−1^ in Fig. 13(c). A linear fit to the data gave a slope of 74 K, which equates to a value of *τ*_0_ ~ 10^−9^ s. Thus, this sample is well described by the Néel-Arrhenius law indicating no magnetic dipolar interactions which is relevant for a typical SPM-type of behavior. In light of the negative Curie-Weiss temperature, we believe that this system consists of AF clusters embedded within a SPM-like matrix.

### Magnetic nanoparticles within random magnetic anisotropy (RMA) model

In order to understand such complex systems, we refer to the model suggested for disordered magnetic nanoparticle systems within the random magnetic anisotropy (RMA) model^28^ with competing dipolar interactions and exchange couplings by Mao *et al*.^29^. The total energy of the system reduced by the anisotropy energy K_*A*_V is written as

6 $$\begin{array}{rcl}E & = & -\sum _{i}{({\hat{s}}_{i}\cdot {\hat{e}}_{i})}^{2}+g\sum _{i,j}\frac{{\hat{s}}_{i}\cdot {\hat{s}}_{j}-\mathrm{3(}{\hat{s}}_{i}\cdot {\hat{r}}_{i,j})({\hat{s}}_{j}\cdot {\hat{r}}_{i,j})}{{r}_{i,j}^{3}}\\  &  & -J\sum _{{r}_{i,j}\le \xi }{\hat{s}}_{i}\cdot {\hat{s}}_{j}-h\sum _{i}{\hat{s}}_{i}\cdot \hat{H}\end{array}$$

Here, magnetic moment of cluster *i* is assumed to be ${M}_{s}V{\hat{s}}_{i}$, where *M*_*s*_ is the saturation magnetization and ${\hat{s}}_{i}$ is the orientation while ${\hat{e}}_{i}$ is the direction of the easy axis with the anisotropy constant K_*A*_. The reduced energies, *g*, *J* and *h* are the dipolar, exchange and Zeeman energies. The distance between the clusters *i* and *j* is given by *r*_*i*,*j*_ in units of *ξ* (${\hat{r}}_{i,j}$ indicates the direction of *r*_*i*,*j*_). The average distance between two nearest-neighbor of clusters is *ξ* for nanospheres of volume V = R^3^.

Under the mean field approximation, due to random anisotropy^28^ the mean anisotropy of the system is zero, which leads to the Curie–Weiss temperature *T*_CW_ = 0. For a ferromagnetic exchange system, the mean field of ferromagnetic exchange coupling is positive, yielding *T*_CW_ > 0. For a disorder (random anisotropy axes) system with dipolar interaction^30^, such as in the present case with *x* = 0.5, a negative mean field is expected from the first part of the dipolar energy $(g\frac{{\hat{s}}_{i}\cdot {\hat{s}}_{j}}{{r}_{i,j}^{3}})$ while the average over the random field $(-g\frac{\mathrm{3(}{\hat{s}}_{i}\cdot {\hat{r}}_{i,j})({\hat{s}}_{j}\cdot {\hat{r}}_{i,j})}{{r}_{i,j}^{3}})$ of the second part of the dipolar energy would give zero, yielding *T*_CW_ < 0. In a competing interaction system, with *x* ≥ 0.5, *T*_CW_ can increase from negative to positive as *J* increases for a fixed value of *g*. When *T*_CW_ < 0, dipolar interactions are dominant where SSG-type phase may appear or dipolar interactions are suppressed and SPM-type phase may appear. With increase in *J*, *T*_CW_ > 0, exchange coupling may dominate and ferromagnetic order can prevail.

**Figure 14:**
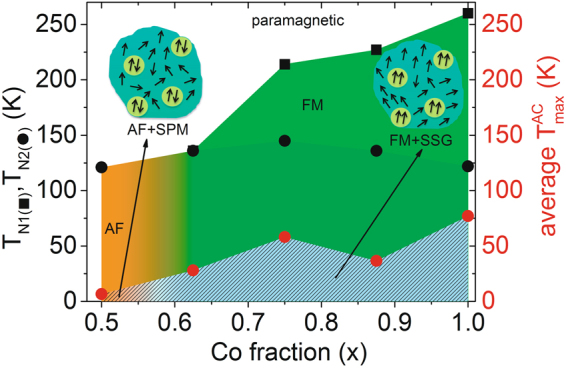
Proposed magnetic phase diagram. A plot of the average ${T}_{{\rm{\max }}}^{AC}$ (red circle) and *T*_N1,N2_ (black symbols) as a function of cobalt fraction delineates the various magnetic phases observed for this system. The shaded region below *T*_N1,N2_ indicate which phases are intra-particle and the one below ${T}_{{\rm{\max }}}^{AC}$ are inter-particle phases. Insets are cartoons of the FM/SSG matrix and AF/SPM matrix phases.

It was reported earlier that by changing the volume density over a wide range, one can monitor the effect of dipolar interactions^31^. In Fig. 14 we sketch the complex magnetic phase diagram deduced from our susceptibility results as function of temperature and Co fraction (*x*) and explain it in terms of competition between the various dipolar, exchange, inter- and intracluster interactions. We plot the change in average ${T}_{{\rm{\max }}}^{AC}$ and *T*_N1,N2_ as a function of cobalt fraction showing the different regimes. One clearly observes an obvious paramagnetic phase at high temperature for all *x*. Below *x* = 0.5, the system is increasingly paramagnetic at all temperatures. Competitive and diluted interactions are induced by the presence of Fe and Co atoms. Thus, large Fe content stabilizes the AF phase while low Fe content (high Co) stabilizes the FM phase.

For *x* = 0.5 (dilute system), the NCs exhibit a two-stage magnetic transition, i.e., an antiferromagnetic phase (*T*_CW_ < 0) at ~121 K and a SPM-like phase with embedded AF clusters at ~6.5 K. The microscopic origin of antiferromagnetism can be due to the presence of small proportions of exchange-coupled antiferromagnetic Co oxide aggregates while the NCs remain isolated. Oxides may include CoO, Co_3_O_4_, etc., with different Neél temperatures of 290 K and 40 K. However, we do not observe any exchange bias even below 40 K. This indicates the presence of randomly associated (decoupled) isolated nanocrystals. Random agglomeration of magnetic domains leads to demagnetizing effects that decreases coercivity. This scenario is consistent with the obtained NC sizes. The NC system is evolving here from a randomly coupled isolated system (*x* < 0.5) to an exchange coupled antiferromagnetic NP system embedded in a SPM matrix (*x* = 0.5). The NPs can acquire a a finite moments due to a canted spin state. Thus, in such a scenario, a competition between the exchange coupling within the antiferromagnetic clusters and the dipolar forces between the clusters is plausible. Once an antiferromagnetic coupling is established, dipolar interactions are no longer possible. Consequently, the dipolar interaction is not sufficiently strong here and a SPM-like phase is established^32^.

For *x* ≥ 0.625, the magnetic dynamics change the two-stage magnetic transitions due to increasing dipolar interaction. The magnetic behavior changes from ferromagnetism (*T*_CW_ < 0) at ~136 K to that resembling SSG-like phases with embedded FM clusters at ~28 K. The basic physical picture is provided by the RMA model encapsulated in equation (6). It describes that as the volume fraction of Co increases, the effects of both dipolar and exchange interactions are evident. The aggregates evolve from isolated NCs to exchange-coupled magnetic nanoparticles. The SSG-like behavior comes from the competition between a mixture of ferromagnetic and antiferromagnetic clusters interacting with each other via dipolar forces.

## Conclusions

We have synthesized cobalt iron pyrite (Co_*x*_Fe_1−*x*_S_2_) nanocrystals and investigated their structure, morphology, and magnetic properties. Using XRD, SAXS, SEM and TEM, we show that there is a systematic variation in the lattice constant, primary grain size, and aggregate size with increasing cobalt content. We find a reasonable agreement of lattice constant *a* with Vegard’s law which is evidence for alloying with *x* close to the nominal ones. TEM-based EDS confirmed these compositions. Each nanoparticle is a collection of a few NCs whose number remain similar over a wide range of *x*.

Magnetically, the cobalt iron pyrites show interesting regimes of competing exchange and dipolar interactions with increasing Fe content, which is investigated using DC magnetization and AC susceptibility at various temperatures and frequencies. Below *x* = 0.5, the system remains mostly paramagnetic. With an increase in *x*, for *x* = 0.5, the nanocrystals remain largely isolated as they show non-interacting SPM-like behavior, *i.e*. absence of magnetic dipole interactions between the embedded AF nanoclusters. Here, the exchange interaction is not strong enough to suppress the SPM behavior. However, dipolar interactions result in a collective state at larger *x*. For *x* ≥ 0.625, the collective state possesses SSG-like behavior along with embedded FM characteristics. In samples of magnetic nanoparticles, exchange interactions are often important and can be sufficiently strong to suppress the SPM relaxation. We have constructed a phase diagram as function of temperature and Co fraction and explained the terms of competition between the various dipolar, exchange, inter- and intracluster interactions.

Functional *n*-doped iron pyrite with tuning possibilities of NC size and shape has opened up new prospects in the field of spin polarized ferromagnetism. In the future, improvements in the NC quality, detailed structural analysis, and analysis of functional properties will be addressed.

## Methods

### Sample preparation

#### Chemicals

Anhydrous iron (II) chloride (98%, Aldrich), anhydrous cobalt (II) chloride (99.7%, Alfa Aesar), sulfur powder (99.998%, Aldrich), chloroform (99.5%, Aldrich), anhydrous ethanol (99.5%, Aldrich), octadecylamine (90%, Acros), phenyl ether (99%, Acros) and argon gas (99.999%, Praxair) were used as received.

#### Nanocrystal Synthesis

Co_*x*_Fe_1−*x*_S_2_ nanocrystals were synthesized using a modified version of a literature recipe^3^. All steps were performed using standard air-free techniques. A total of 3 mmol of MCl _2_ (M = Co + Fe, with the ratio of the two metals determined by the desired Co_*x*_Fe_1−*x*_S_2_ NC compositions of *x* = 0.0, 0.125, 0.25, 0.375, 0.5, 0.625, 0.75, 0.875, and 1.0) was dissolved in 25 g of octadecylamine at 80 °C, then degassed under vacuum for one hour. Separately, 1 mmol of sulfur was dissolved in 10 mL phenyl ether at 90 °C, then vacuum degassed for one hour. After degassing, the MCl_2_ solution was heated to 215 °C, whereupon the sulfur solution was quickly injected into the MCl_2_ solution to initiate nanocrystal nucleation. The reaction was held at 218 °C for 3 hours, then quenched in a water bath and diluted with 20 mL of ethanol once the temperature fell to 95 °C. The raw product was collected by centrifuging the reaction mixture for 3 min, removing the supernatant, and resuspending the solid in 30 mL of chloroform. Two additional rounds of centrifugation and reprecipitation were used to clean the nanocrystals. The purified nanocrystals were dried and stored as a loose powder.

### SEM and TEM

The local morphology of the NCs were imaged using a FEI Magellan 400 XHR scanning electron microscope (SEM) operating at 10 kV (50 pA) and an FEI-Philips CM20 transmission electron microscope (TEM) operating at 200 kV.

Co:Fe ratios were measured by energy dispersive X-ray spectroscopy (EDS) in a JEOL JEM-ARM300F GrandARM (S)TEM equipped with dual silicon drift detectors (0.98 sr collection solid angle) and operating at 300 kV. Co_*x*_Fe_1−*x*_S_2_ NCs were dispersed in chloroform (10 mg/mL) with 5 minutes of sonication. Copper TEM grids with carbon film coatings (400 mesh, Ted Pella) were dipped into the NC solution for 3 s, rinsed with chloroform and anhydrous ethanol to remove unreacted precursors, and dried. EDS maps of four large particle clusters (>500 particles each) were collected for each sample after a 20 minute electron beam shower. Quantitative EDS analysis utilized the Cliff-Lorimer model as implemented in the Gatan Microscopy Suite software package.

### X-ray characterization: XRD

Powder X-ray diffraction (XRD) measurements were performed on a Rigaku SmartLab X-ray diffractometer in Bragg-Brentano reflection geometry with a 2*θ* angular range of 20 to 90 degrees.

### X-ray characterization: SAXS

In order to get a statistical information about the morphology of the NCs powders, we carried out small-angle X-ray scattering (SAXS) measurements. SAXS was performed on a Ganesha 300XL instrument (SAXSLAB ApS, Copenhagen/Denmark) equipped with a GENIX 3-D microfocus Cu X-ray source operating at 50 kV/0.6 mA (wavelength *λ* = 1.542 Å). The samples were mounted in between adhesive tapes. A two-dimensional Pilatus 300 K detector was used, which can be moved to the desired sample-to-detector distance for WAXS or SAXS resulting in a *q*-range of 0.01–0.25 nm^−1^ where *q* is the momentum transfer corresponding to the scattering angle 2*θ* via *q* = 4 *πsin*(*θ*)/*λ*. A pin–diode detector was used to record the beam intensity and transmission of each sample and of the sample holder. All images were corrected for cosmic background and parasitic scattering. The obtained 2-D images were azimuthally integrated to get 1-D data, and the background from the adhesive tapes was subtracted prior to data analysis.

For analysis, the raw data was converted to intensity versus momentum transfer *q* with the software DPDAK (v.0.2.9) using a sample to detector distance of 1056 mm and a detector pixel size of 172 micron^9^. The SAXS data were fit with the software Genplot (v.2.11 by Computer Graphic Service Ltd.).

SAXS data are modelled using the crude approximation for dense system known as LMA which is often used to describe a polydispersed system by separating the form factor from the interference function. The LMA hypothesis assumes that the system is comprised of locally monodispersed domains that interfere incoherently. The particle-particle pair correlation function can vary from domain to domain. The surrounding of each particle is supposed to be made of particles of same size and shape in such a way that the particle kind varies slowly across the sample but with a spatial wavelength lower than the coherence length of the beam. The total scattering intensity is obtained by an incoherent sum of the intensities from each domain of monodisperse subsystems weighted according to the size-shape distribution and is given by

7 $$I(q)={\langle |F(q,R){|}^{2}\ast S(q,R)\rangle }_{D}$$

where * denotes the convolution product and 〈...〉_*D*_ is the average over coherent domain D enclosing the form factor |F(*q*, R)|^2^ in which the local interference function S(*q*, R) can depend on the particle size^15^.

Features in scattering data at low *q* values arise from instrumental resolution effects. To fit the full scattering curve, these contributions from the instrumental resolution are modelled by considering a Lorentzian function. The intensity maximum provide a good estimate of the particle size. The smeared intensity minima of the SAXS data indicate a modest polydispersity in size distribution. Two form factors of the form

8 $$|F(q,R){|}^{2}=\mathrm{9[}\sin \,(qR)-qR\,\cos \,(qR{)]}^{2}/{(qR)}^{6}$$

with spherical geometry distributed over a 1-D paracrystalline^14^ lattice were considered. These form factors correspond to radii R and R′ of the scattering objects. The proportion of spherical particles with radius R′ was very small in comparison to the spherical particles with radius R. A Gaussian distribution function is used to describe the polydispersity of the scattering objects and is given by

9 $$G(R)=\mathrm{1/(}\sqrt{2\pi }\Delta R)\exp [-{(R-\langle R\rangle )}^{2}\mathrm{/(2}\Delta {R}^{2})]$$

where 〈R〉 and ΔR represent the mean and the distribution of the particle radii. Notably, the size distribution of the NPs are often difficult to determine precisely, and are typically assumed to be spherical with log-normal size distributions.

The distribution of particles is given by the inter-particle correlation or local interference function S(*q*,R). The structure factor S(*q*,R), on omitting the homogeneous part, is expressed with the pair correlation function g(R) by

10 $$S(q)=1+{n}_{P}\int [\,g(R)-\mathrm{1]}\exp [iq\cdot R]dR$$

where n_*P*_ is the number density of particles. According to paracrystal theory, the scattering function is affected by the shape of the aggregation and expressed as a convolution product of S(*q*)*γ(*q*) where *γ*(*q*) is the structure factor of the aggregation by which finite size effects are introduced. The respective correlation functions of *γ*(*q*) (or the Debye–Bueche equation) and S(*q*) are known and are given by

11 $$\gamma (q)=\exp [-\frac{R}{\xi }]$$

and

12 $$S(q)=(1-\exp \,[-{q}^{2}{\sigma }^{2}])/[(1+\exp \,[-{q}^{2}{\sigma }^{2}]-2\exp \,[-\frac{1}{2}{q}^{2}{\sigma }^{2}]\cos \,(q,\xi )]$$

where *σ* and *ξ* are respectively the square-root of the variance and the mean value of the the distance probability or the correlation length^15^. S(*q*)*γ(*q*) is obtained by taking the Fourier transform of the products of the correlation functions. Thus the average center-to-center distances (*ξ* and *ξ*′) of the scattering objects (R and R′) were associated with the structure factors and were also obtained from the fits. The distributions of radii (ΔR) and standard deviations of correlation lengths (*σ*) were also used as fit parameters. The SAXS signal from the whole sample volume revealed an isotropic scattering pattern from the pyrites which means there was no azimuthal dependence of the signal on the 2-D detector.

### Magnetometry

Conventional in-plane magnetization measurements were performed as a function of temperature and field using a superconducting quantum interference device (SQUID) from Quantum Design (MPMS-XL). Conventional AC field susceptibility measurements were acquired at various temperatures and frequencies using a physical property measurement system (PPMS).
